# Lower SARS-CoV-2 household transmission in children and adolescents compared to adults

**DOI:** 10.1038/s41598-022-24643-2

**Published:** 2022-12-27

**Authors:** L. Schumm, J. Blankenburg, E. Kahre, J. Armann, A. H. Dalpke, C. Lück, R. Berner, P. Czyborra

**Affiliations:** 1 Department of Paediatrics, University Hospital and Medical Faculty Carl Gustav Carus, Technische Universität Dresden, Dresden, Germany; 2 Institute of Medical Microbiology and Virology, University Hospital and Medical Faculty Carl Gustav Carus, Technische Universität Dresden, Dresden, Germany

**Keywords:** Infectious-disease diagnostics, Virology

## Abstract

In the COVID-19 pandemic, children were considered to play a major role in SARS-CoV-2 transmission similar to influenza. Thus, mitigation measures have been focused on children, impacting their everyday life severely. Despite this, infectivity in this age group regarding SARS-CoV-2 is not yet clarified. We performed a serology study in households with confirmed SARS-CoV-2 infection to evaluate virus transmission with focus on children and adolescents. Between January and July 2021, 341 minors and 650 adults from 300 households with a confirmed index case participated in the FamilyCoviDD19-study including serological assessment for SARS-CoV-2 antibodies and a questionnaire on demographics, recent and ongoing symptoms, hygiene measures and comorbidities. 45 (16.3%) of all index cases were < 18 years old. Thereof, 55.6% reported COVID-19 associated symptoms, while nearly all adult index cases were symptomatic (94.8%). There was significantly less virus transmission by children and adolescents compared to adult index cases with a secondary attack rate of 0.29 vs. 0.54. With the caveat that the results do not necessarily apply to the Delta and Omicron variants, we conclude that children and adolescents are less susceptible for SARS-CoV-2 infection, more frequently show an asymptomatic course of disease and are less infective than adults.

## Introduction

Since the emergence of the novel Severe Acute Respiratory Syndrome Coronavirus 2 (SARS-CoV-2) and the beginning of the pandemic^1^, various mitigation measures have been implemented to contain the spread of the virus. As children often show pauci- or asymptomatic disease, which results in undetected infections^2,3^, it was widely assumed that children are more likely to spread the coronavirus. Similar to the influenza epidemics^4^, children and adolescents were considered to play a leading role in the COVID-19 pandemic and thus strong measures e.g. school closing and frequent testing have been implemented. All of which has specifically impacted this age group. Restricting access of public spaces to those who are fully recovered, vaccinated or otherwise tested has further deprived children of participation in society. It has been shown, that social isolation during the pandemic affects the mental health of children and adolescents, increasing the risk of anxiety and depression^5–7^. At the same time the limited access to public spaces created growing social pressure on children to get vaccinated although the benefit-risk ratio for healthy children is still under debate^8–10^. However, since there is little data on the infectivity of children and adolescents, there exists limited evidence for the justification of the prevention strategies and the vaccination recommendations that apply to minors. To strike a balance between the containment of the pandemic and child’s education and mental health, there is a need for infection control measures that are both highly effective and unrestrictive as possible.

We conducted a seroprevalence study in households with at least one confirmed case of SARS-CoV-2 to determine the transmission rates within a setting of close human contact with a focus on children and adolescents. This was done to characterize the susceptibility and infectiveness towards the novel coronavirus and to further define risk factors of virus transmission. Moreover, we evaluated the effectiveness of specific hygiene measures implemented in the household during quarantine as well as risk factors for prolonged symptoms.

## Results

**Figure 1:**
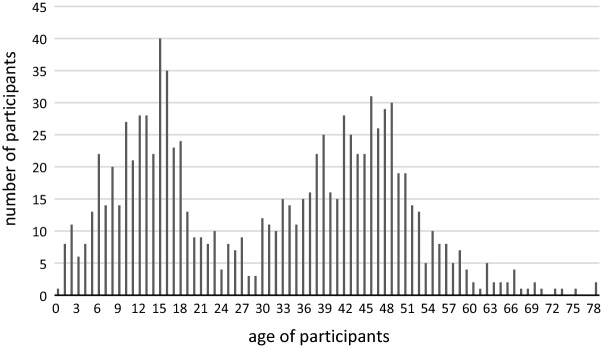
Age distribution of the study population. The number of participants for every age from 0 to 79 years is displayed. Minimum age was 0 years and maximum age was 79 years. Median (IQR) age of all participants was 33 (15–46).

Three hundred households with a total of 1056 household members and a median [IQR] household size of 4 [3–5] members were enrolled in the study. 991 (94%) of all household members participated in the study. Median age of the participants was 33 [15–46] and 341/991 (34.4%) were < 18 years of age (see Fig. 1 for the exact age distribution). In total, 950 participants consented to blood collection and were tested for antibodies against SARS-CoV-2. Of the 950 participants, 587 (61.8%) were seropositive. An index case was identified in 276/300 (92%) households. 244 (88.4%) of all index cases reported a symptomatic infection with a significant difference between index cases < 18 years old and index cases ≥ 18 years old (55.6% vs 94.8%; p < 0.0001). The ratio of index cases < 18 (16.3%) and ≥ 18 years old (83.7%) was also clearly different. 229/276 (83.0%) index cases were seropositive in our analysis. This ratio was the same for index cases < 18 and ≥ 18 years of age. 370 (63%) of all seropositive participants reported a previous positive SARS-CoV-2 PCR. In addition, participants with comorbidities were more likely to be seropositive (68.3% vs 55.9%, p < 0.001) and to have symptomatic SARS-CoV-2-infections (69.4% vs 56.4%; p < 0.0001) compared to those without. Further demographics of the study population are listed in Table 1.

**Table 1:** Descriptive statistics of the study population.

Households, total	300	
Household size, median (IQR)	4	(3–5)
Household members, total	1056	
**Study participants**	991	
≥ 18 years	650	(66%)
< 18 years	341	(34%)
**Age, median (IQR)**	33	(15–46)
≥ 18 years	43	(34–49)
< 18 years	11	(7–15)
Sex, male	482	(49%)
Days from infection to serology, median (IQR)	82	(59–111)
**Households with Index Case**	276	
Index ≥ 18 years	231	(84%)
Seropositive	194	(84%)
Symptomatic	219	(95%)
Index < 18 years	45	(16%)
Seropositive	35	(78%)
Symptomatic	25	(56%)
**Symptomatic COVID-19**		
Participants with comorbidities	197	(69%)
Participants without comorbidities	390	(56%)

Regarding transmissions within the households, in 27/299 (9%) households none of the participants were seropositive and in 58/299 (19.4%) households only the index-case was seropositive. In 112/299 (37.5%) households all household members were seropositive. There was a lower chance that everyone in the household was seropositive with a minor index case than with an index case ≥ 18 years (18.2% vs 43.8%; p = 0.0013) (Table 2). Accordingly, the SAR of underage index cases was significantly lower compared to ≥ 18 years old index cases (0.29 vs 0.54; p < 0.0001) (Table 3).

**Table 2:** Household contagion after SARS-CoV-2-infection: according to different household characteristics.

Households [%]	All households	With < 18 y/o	Without < 18 y/o	Index < 18 y/o	Index ≥ 18 y/o	Symptomatic Index	Asymptomatic Index	2 members	3 members	4 members	> 4 members
Everyone seropositive	37.6	22.2	61	18.2	43.8	43.9	0	63.9	36.4	23.3	17.6
Some seropositive	34.2	47.7	14.4	29.5	30.5	33.5	10.5	5.6	36.4	50	70.6
Only index seropositive	19.7	21.6	16.1	38.6	17.7	16.7	42.1	18.1	20	20	5.9
no one seropositive	8.5	8.5	8.5	13.6	8	5.9	47.4	12.5	7.27	6.7	5.9
		χ^**2**^ (3, 294) = 52.18; p < 0.00001		χ^**2**^ (3, 270) = 15.09; p = 0.0017		χ^**2**^ (3, 258) = 51.37; p < 0.00001		χ^2^ (9, 231) = 58.09; p < 0.00001			

Note: Chi Square was used for statistical analysis.

**Table 3:** SARS-CoV-2 secondary attack rates (SAR).

Overall	0.53
**Index case**	
< 12 years	0.38
12–18 years	0.26
< 18 years	0.29*
18–40 years	0.44
> 40 years	0.60
≥ 18 years	0.54
**Index < 18 years**	
Contacts < 18 years	0.29
Contacts ≥ 18 years	0.29†
**Index ≥ 18 years**	
Contacts < 18 years	0.48
Contacts ≥ 18 years	0.59
**Index case**	
Symptomatic	0.58
Asymptomatic	0.02*
**Household**	
Aware of infection	0.52
Unaware of infection	0.22ˣ

Note: Fisher’s exact test was used for statistical analyses.

*p < 0.0001 compared to index cases ≥ 18 years.

†p = 0.017 compared to index ≥ 18 years to contacts  <18 years.

٭p < 0.0001 compared to symptomatic index cases

ˣp < 0.0001 compared to households aware of infection.

Specifically, transmission from adult index cases to household contacts < 18 years was more common than vice versa (SAR 0.48 vs 0.29; p = 0.017). Children and adolescents generally transmitted SARS-CoV-2-infections less frequently to both adult and underage household contacts (SAR 0.29). When comparing transmissions within households with PCR confirmed SARS-CoV-2-infections to those in which the SARS-CoV-2-infection was detected only retrospectively by serology, we found that SAR was significantly higher within households with a positive PCR (0.52 vs 0.22; p < 0.0001). Even when a household did not follow any hygiene measures during quarantine, SAR was higher than in households that were unaware of their SARS-CoV-2-infection (0.53 vs 0.22; p < 0.0001). Accordingly, in households with a symptomatic index person there was a higher chance that every participant from the household was seropositive (44.1% vs 0%; p < 0.0001). The seropositivity rate was also dependant of the household size as it decreased significantly for each additional household member. In households with more than 4 members the probability of all household members being seropositive was significantly lower compared to households with only two members (17.6% vs. 63.9%; p < 0.0001).

### Effect of hygiene measures

To analyse the effect of hygiene measures on transmission, participants were asked if they were following specific hygiene measures e.g. physical distancing, wearing of masks or regular room ventilation. Interestingly, the overall SAR was the same in households that implemented hygiene measures to those that did not (0.53) (Table 4). When the index case left the household (6/211 households) as soon as the infection was confirmed, there was a 33.3% chance that no other household member was seropositive (SAR of 0.36), whilst this was only the case in 15% of the households without any measures (SAR 0.53). Due to the low case numbers, this was not statistically significant.

**Table 4:** Household contagion after SARS-CoV-2-infection: according to hygiene measures.

Households [%]	No hygiene measures	Unaware of infection	Any hygiene measures	Index moved out	Separate rooms	Separated use of rooms	Physical distance	Hand hygiene	Air ventilation	Face mask
Everyone seropositive	42.5	7.5	43.4	16.7	27	40.7	42.9	46.7	46.1	30
Some seropositive	27.5	45	33.2	33.3	37.8	33.3	34.8	31.5	31.8	32
Only index seropositive	15	40	16.1	33.3	24.3	16.7	13.4	15.8	14.3	24
noone seropositive	15	7.5	7.3	16.7	10.8	7.4	6.3	6.1	7.8	10
		χ^**2**^ (3, 80) = 17.04 p < 0.001	χ^**2**^ (3, 251) = 2.67 p = 0.45	χ^**2**^ (3, 46) = 1.97 p = 0.58	χ^**2**^ (3, 77) = 3.06 p = 0.38	χ^**2**^ (3, 94) = 1.54 p = 0.67	χ^**2**^ (3, 152) = 3.11 p = 0.37	χ^**2**^ (3, 209) = 3.60 p = 0.31	χ^**2**^ (3, 199) = 2.07 p = 0.56	χ^**2**^ (3, 90) = 2.43 p = 0.49

Note: Chi Square was calculated compared to households with no hygiene measures.

### Long-term effects of COVID-19

To evaluate the severity and long-term effect of the infection, participants were asked about current medical conditions at the time of blood collection. Results are displayed in Table 5 as ongoing symptoms < 12 weeks and ≥ 12 weeks after SARS-CoV-2 infection and analysed according to specific characteristics of the participants. When the infection was < 12 weeks ago 25% of the participants suffered from ongoing symptoms while participants that were enrolled ≥ 12 weeks after their infection reported health restrictions in 29.7%. Seropositive participants were more likely to have persistent symptoms both < 12 and ≥ 12 weeks after infection within their household, but interestingly, seronegative participants also reported ongoing symptoms (< 12 weeks: 31.5% vs 14.5% and ≥ 12 weeks: 34.8% vs 20.8%; p < 0.001). When analysing only participants < 18 years of age, differences in ongoing symptoms between seronegative and seropositive participants were not significant. Individuals that reported SARS-CoV-2 related symptoms in the acute phase were more often suffering from ongoing symptoms than asymptomatic participants both < 12 and ≥ 12 weeks after the event of SARS-CoV-2-infection within the household (< 12 weeks: 33.3% vs 5.0% and > 12 weeks: 39.8% vs 3.1%; p < 0.0001).

**Table 5:** Ongoing symptoms after SARS-CoV-2-infection.

Time after SARS-CoV-2	Ongoing symptoms	Male	Female	< 18 years	≥ 18 years	< 12 years	12–17 years	18–40 years	> 40 years
< 12 weeks	Yes	19.7	30.1	8.2	32.6	6.3	10.8	21.3	40.6
	No	80.3	69.9	91.8	67.4	93.8	89.2	78.7	59.4
		n = 132	n = 136	n = 85	n = 181	n = 48	n = 37	n = 75	n = 106
		p < 0.05		p < 0.0001		ns		p < 0.01	
≥ 12 weeks	Yes	17.5	40.4	3.8	41.9	0	7.3	37.8	45.5
	No	82.5	59.6	96.2	58.1	100	92.7	62.2	54.5
		n = 160	n = 183	n = 105	n = 234	n = 50	n = 55	n = 111	n = 123
		p < 0.0001		p < 0.0001		p < 0.01		p = 0.053	

Time after SARS-CoV-2	Ongoing symptoms	Seropositive	Seronegative	Symptomatic COVID-19	Asymptomatic COVID-19	Comorbidities	No comorbidities	BMI < 25	BMI > 25*
< 12 weeks	Yes	31.5	14.5	33.3	5	42.5	17.8	8.2	0
	No	68.5	85.5	66.7	95	57.5	82.2	91.8	100
		n = 168	n = 83	n = 189	n = 80	n = 80	n = 185	n = 73	n = 5
		p < 0.001		p < 0.0001		p < 0.0001		ns	
≥ 12 weeks	Yes	34.8	20.8	39.8	3.1	41	24.5	2.2	22.2
	No	65.2	79.2	60.2	96.9	59	75.5	97.8	77.8
		n = 233	n = 101	n = 244	n = 98	n = 100	n = 241	n = 91	n = 9
		p < 0.001		p < 0.0001		p < 0.01		p < 0.05	

Note: table shows fraction of participants with and without ongoing symptoms [%] with the total number of participants per column, p-values refer to Fisher’s exact test results.

*BMI analysis for participants < 18 years old only.

Generally, adults were more likely to develop ongoing symptoms compared to children and teenagers < 18 years (< 12 weeks: 32.6% vs 8.2%; p < 0.0001 and ≥ 12 weeks: 41.9% vs 3.8%; p < 0.0001). Furthermore, participants > 40 years of age were more likely to have ongoing symptoms up to 12 weeks after infection than young adults (46.9% vs 21.3%; p < 0.01). When participants ≥ 18 years were enrolled ≥ 12 weeks after the infection, they more often reported medical issues than those seen within 12 weeks after infection (41.9% vs 32.6%) while the rate of continuing symptoms in minor participants decreased over time (< 12 weeks 8.2% vs ≥ 12 weeks 3.8%). The probability of ongoing symptoms did not only correlate with age but with sex. Female participants had a higher prevalence of ongoing symptoms ≥ 12 weeks after the infection compared to male participants (40.4% vs 17.5%; p < 0.0001). Consistent with higher infection rates and seroprevalence, participants with comorbidities were more likely to experience persisting symptoms after the infection (< 12 weeks: 42.5% vs 17.8%; p < 0.0001 and > 12 weeks: 41.0% vs 24.5%; p < 0.01). Here, seropositive participants were significantly more affected than seronegative subjects (47.5% vs 21.6%; p < 0.005). Comorbidities have been reported by 284/991 (28.7%) participants and included hypertension and hypothyreosis most frequently. Specifically, 57/341 (16.7%) of children and adolescents had comorbidities of which atopic diseases accounted for most chronic diseases. BMI did not have any influence on the rate of ongoing symptoms in participants ≥ 18 years old, but children and adolescents with a BMI > 25 were more likely to report ongoing symptoms ≥ 12 weeks after the infection than those of lower BMI (22.2% vs 2.2%, p < 0.05).

## Discussion

In this prospective seroprevalence study, we analysed SARS-CoV-2-transmission in 300 households with confirmed index-cases to evaluate infectivity and susceptibility in a setting of close contact. We found that 587/950 (61.8%) household members were seropositive after an event of SARS-CoV-2-infection within their household. This rate is higher than in a previous analysis from January 2021^11^ and most other studies^12–14^. While the incident rate was the same in children and adults in the household setting, only 16.3% of the index cases were < 18 years old, indicating that SARS-CoV-2-infections were mostly introduced into the household by an adult household member. Additionally, the SAR was much lower for underage index cases compared to that of adult index cases. Children and adolescents were less likely to infect both household contacts of the same age and adult household contacts (SAR 0.29), which indicates a lower infectivity in this age group. This is in line with several previous studies^15–17^ while some others found children to be more likely than adults to transmit the virus^18^. However, overall, SARS-CoV-2 transmission rates from children seem to be lower in contrast to other respiratory viruses, for which children were found responsible for most of transmission clusters^19^. In addition to the lower SAR in children and adolescents in our study, participants in this age group showed fewer symptoms during infection and were more often asymptomatic compared to adults which is very consistently described in literature^2,20,21^. This indicates that an asymptomatic SARS-CoV-2-infection is associated with a reduced infectivity and contradicts the assumption that children quickly spread the virus because of asymptomatic and therefore undetected infections. This evidence is in line with observations in other studies^22^. In households, that were enrolled only after positive seroanalysis of a child or adolescent within the SchoolCoviDD19-study and did not know of any event of SARS-CoV-2-infection within their household, the SAR was the lowest of all (SAR 0.22), which supports this theory.

In contrast to other studies, we did not identify any hygiene measures that were associated with a reduced seropositivity rate within the household. This seems logical considering that the infectivity is highest even before and until shortly after the first symptoms^23,24^ while hygiene measures are implemented only when the infection is PCR-confirmed a few days later. Wang et al. found combined hygiene measures to only be effective when implemented before the beginning of symptoms^25^. Therefore, they serve as a preventative measure rather than a mechanism used after detection of the virus. Otherwise, the lack of effect of the hygiene measures might also indicate a higher contagiousness of the alpha variant since similar previous studies could detect an effect.

Sometime after the start of the pandemic, discussions emerged about ongoing or recurring symptoms after the initial SARS-CoV-2-infection, the so-called Long-COVID-syndrome. In this study we screened for the presence of any symptom at the time of the study visit and found that nearly every third participant reported any ongoing symptom. These symptoms were clearly associated with a symptomatic SARS-CoV-2-infection and with a positive serostatus. However, it is important to mention, that 20.8% of the seronegative participants also reported symptoms ≥ 12 weeks after COVID-19 within their household. Even though there is no seroconversion in about 10% of people after a SARS-CoV-2 infection^26,27^, these results show that there is a certain background incidence of medical conditions that is not related to the virus infection. Part of it most likely represents a baseline level of non-specific symptoms within our population which might be elevated due to the restrictions during the pandemic. Furthermore there is evidence, that ongoing symptoms cluster within households^28^. Seronegative but exposed individuals were more likely to experience ongoing symptoms when infected household members reported ongoing symptoms. This phenomenon should be considered when patients present Long-COVID-like symptoms. This is important to not over- or misdiagnose, and to involve other household members into the therapy. Our findings suggest that the rate of Long-COVID-syndrome is overestimated as many studies on Long-COVID did not include seronegative control groups and have thus not taken into account the baseline prevalence of ongoing symptoms^29,30^. In line with the milder course of COVID-19 in children and adolescents, they were least often suffering from ongoing symptoms while the rate of prolonged disease increased with age. More importantly, in contrast to adults, there was no significant difference in the rate of ongoing symptoms between seropositive and seronegative children and adolescents. In our SchoolCoViDD19-study we recently found the same results when screening 1560 students for Long-COVID-symptoms, which again suggests an overestimation of Long-COVID-syndrome within < 18 year olds and provides further evidence of a significant negative effect of the infection control measures during the pandemic^7^. Our study detected comorbidity as a risk factor for ongoing symptoms and this was specifically associated with the SARS-CoV-2-infection as seronegative participants with comorbidities suffered significantly less often from ongoing symptoms. Previous studies showed that common medical conditions e.g. diabetes or hypertension are risk factors for severe COVID-19 and increased mortality^31^.

### Limitations

We did not screen households for SARS-CoV-2 infection with the agenda of recruiting them for our study. Rather households were invited to participate in our study upon proof of SARS-CoV-2 infection of at least one household member, no matter their reason for the testing. This may influence the representation of index cases in our study and may be dependent on current testing strategies. However, an index case was not necessarily defined by a positive PCR or antibody result but beginning of symptoms was also considered. Moreover, 44.9% of the participants reported that PCR tests were done because of symptoms and 32.5% had been tested because of a positive contact. Only 4.1% of the tests were done as a routine testing at work or in school, which reveals that index case detection was not dependent of the public testing strategy in our study.

Screening for Long-COVID-syndrome in a questionnaire is always vulnerable to bias. To minimize the recall bias in our study, we did not specifically mention Long-COVID-symptoms but asked if any symptoms were present at the time of the study visit. This was done before the participants knew about their serostatus. A certain bias in the ongoing symptoms of young children cannot be excluded as parents may have helped with filling the questionnaire.

The results of our study are related to infections at the time the Wuhan and alpha variant were the predominant SARS-CoV-2 variants. Therefore, different virus characteristics should be considered when comparing this study to recent studies. Since there is a higher transmissibility of the omicron variant, the overall SAR will likely be higher than with the alpha variant. However, differences in transmission between children and adults should remain similar as shown recently by Lyngse et al.^32^. At the same time, the virulence of the omicron variant is significantly lower than that of alpha, which may affect the incidence of ongoing symptoms.

## Conclusion

In line with previous studies and clinical reports, we could show that children and teenagers of < 18 years of age did not only have a milder course of COVID-19^33^, but they were less prone to be infected with SARS-CoV-2, and most importantly, less likely to transmit it to their household members. Several other studies found that children and adolescents have a similar seroprevalence for SARS-CoV-2 as adults^34^ and some even reported that the infectivity seems to be reduced^35^, but still most conclude that because of the same susceptibility as adults plus the undetected cases, strict hygiene measures and surveillance should be implemented to prevent SARS-CoV-2-spreading in day cares and schools. However, our study could not confirm an overrepresentation of children and adolescents in SARS-CoV-2 cases^36^ but also shows that their probability to transmit the virus is much lower than that of adult index cases. Consequently, the focus of pandemic control should be directed more towards adults than children and adolescents.

## Methods

Households with a confirmed SARS-CoV-2-infection were contacted by the local Health Department and invited to participate in the FamilyCoviDD19-study in order to investigate SARS-CoV-2 seroprevalence and to define possible risk factors for infection, transmission and prolonged symptoms. The study was approved by the ethics committee of the Technical University of Dresden (BO-EK-342072020) and was performed according to the declaration of Helsinki. Upon informed consent from all participants or their legal guardians, a blood sample was taken from the participants and information about their demographics, past episodes of illness, PCR-testing for SARS-CoV-2, hygiene measures during quarantine as well as their current state of health was obtained using a questionnaire (which can be found online as Supplementary Methods). Participants were asked to fill in the questionnaire before the blood sampling to avoid bias by the result of the seroanalysis.

### Cohort description

Between 18th January and 20th July 2021, 394 households were enrolled into the seroprevalence FamilyCoviDD19 study. Upon a positive SARS-CoV-2-PCR test, 254 households were enrolled whilst 46 were enrolled after positive antibody testing in our seroprevalence SchoolCoviDD19- and KiTaCoviDD19-study^37,38^. Eventually, 21 households were excluded because they did not meet the inclusion criteria (no other household member of the index person participated or household members did not have any contact during the infection period). A further 73 households were excluded in this analysis because at least one household member was vaccinated for SARS-CoV-2. This was done to exclude bias in the calculation of the secondary attack rate by vaccination based seropositivity.

### Seroanalysis

The detection of antibodies against SARS-CoV-2 was performed with the Diasorin LIASON SARS-CoV-2 S1/S2 IgG Assay. If equivocal or positive, the result was confirmed with a second assay, either the Abbott Diagnostics ARCHITECT SARS-CoV-2 or the Euroimmun Anti-SARS-CoV-2 ELISA. The participants’ serostatus was deemed positive, if at least 2 of 3 tests were positive.

### Epidemiological situation

The study visits took place between January and July 2021, the participants’ infections, however, date to a period from October 2020 to May 2021 with a clear peak in December 2020 and January 2021 representing Germany's second COVID-19 infection wave. The 7-day-incidence rate in Saxony ranged from 120.1 to 444.4/100.000 during this period. In terms of the predominant virus variant, this period represents a transition from the original Wuhan variant to the alpha variant (B1.1.7). During this period, Saxony was in strict lockdown which included a curfew and complete closing of schools, kindergardens, restaurants and shops that did not serve basic supply. There was no regular mandatory testing for any part of the population. Wearing of medical face masks was mandatory in supermarkets and public transport.

### Definitions and variables of interest

For every household, one index case was determined if possible. An index case was defined as the person who was most probable the first one infected according to onset of symptoms, positive test or serological testing. In a first approach, to evaluate the transmission within the households, we classified households according to the serostatus of their household members. They were categorized according to whether no household member was seropositive, only the index case was seropositive, some household members were seropositive or all household members were seropositive. For a more specific analysis we further calculated the secondary attack rate (SAR) as a function of seropositive contacts out of all contacts of the index person within the household. Finally, all participants were asked for any health restrictions at the time of the study visit to evaluate the prevalence of ongoing symptoms after COVID-19. Their responses were analysed based on the time interval between the infection/quarantine and the study visit and classified as “< 12 weeks” or “  12 weeks”^39^.

### Data analysis and statistics

All data was managed, and calculations were done in Excel. Statistical analysis was done in GraphPad Prism v9 using Fisher’s exact test for 2 × 2 contingency tables and Chi Square for 2 × 4 contingency tables as indicated.

## Supplementary Information

Supplementary Information.

## Data Availability

The data that support the findings of this study are available from the corresponding author upon reasonable request.
