# Supplementary material for: Lower SARS-CoV-2 household transmission in children and adolescents compared to adults
Source: Sci Rep. 2022 Dec 27;12:22453. doi: 10.1038/s41598-022-24643-2 (PMC9794106; doi:10.1038/s41598-022-24643-2)
Supplement: Supplementary file 1 — Supplementary Information. [file 41598_2022_24643_MOESM1_ESM.pdf]

Lower SARS-CoV-2 household transmission in children and adolescents compared to adults

Schumm L.[1]*, Blankenburg J.[1], Kahre E.[1], Armann J.[1], Dalpke AH.[2], Lück C.[2], Berner R.[1], Czyborra P.[1]

[1] Department of Paediatrics, University Hospital and Medical Faculty Carl Gustav Carus, Technische Universität Dresden, Dresden, Germany

[2] Institute of Medical Microbiology and Virology, University Hospital and Medical Faculty Carl Gustav Carus, Technische Universität Dresden, Dresden, Germany

***Corresponding author:**

Leonie Schumm

leonie.schumm@uniklinikum-dresden.de

Tel: +49 0351 458 0

Fax: +49 351 458 7205

Supplementary Material

Supplementary Methods:

Questionnaire for participants of the Family-CoViDD19-Study (English translation)

University Clinic Carl Gustav Carus

FamilyCoViDD19-Study

Questionnaire

**Study-ID** (*filled in by study team*):		
**age:**	**gender:** ☐ m ☐ f ☐ d	**number of household members:**
**height:** ______ **cm**	**weight:** ____________**kg**	**age of household members:**

For professionals - Profession:

For students - Type of school:

For kids that attend kindergarden – Did the kid regularly attend kindergarden during winter (20/21) (exceptional care during lockdown)? ☐ Yes ☐ No

**Do you have comorbidities?**	**Do you take regular medication?**
☐ Yes, the following:	☐ Yes, the following:
☐ No	☐ No

Have you been tested for SARS-CoV-2?

☐ Yes: ☐ PCR ☐ Antigen-Rapid-Test

When and where? ____________________________

Reason for testing: ____________________________________

Result: ☐ positive ☐ negative

☐ No

Have you been at work/ at school/ in kindergarden 48h before beginning of symptoms/ before SARS-CoV-2 testing?

☐ Yes ☐ No

University Clinic Carl Gustav Carus

FamilyCoViDD19-Study

Have you been sick since October 2020?

☐ No

☐ Yes, with COVID-19 (positive test) or during quarantine (positive test of household member)

☐ I was the first one with symptoms

☐ I had the following symptoms:

- ☐ cough
- ☐ rhinitis
- ☐ sore throat
- ☐ fever
- ☐ headache
- ☐ nausea /vomitting
- ☐ dyspnea
- ☐ muscle / limb pain
- ☐ diarrhea
- ☐ smell / taste disorders
- ☐ fatigue
- ☐ other:

☐ Yes, at another time point than COVID-19 / quarantine (month/year):

☐ I was the first one with symptoms

☐ I had the following symptoms:

- ☐ cough
- ☐ rhinitis
- ☐ sore throat
- ☐ fever
- ☐ headache
- ☐ nausea /vomitting
- ☐ dyspnea
- ☐ muscle / limb pain
- ☐ diarrhea
- ☐ smell / taste disorders
- ☐ fatigue
- ☐ other:

Did you see a doctor within 14 days after SARS-CoV-2 test: ☐ Yes: ☐ in clinic ☐ in hospital

☐ No

Duration of quarantine:

University Clinic Carl Gustav Carus

FamilyCoViDD19-Study

Which hygiene measures have been implemented in the household during quarantine?

**Complete separation (e.g. index person moved out)**	☐ Yes	☐ No
**Temporally seperated use of rooms**	☐ Yes	☐ No
**Physical distance**	☐ Yes	☐ No
**Hand hygiene**	☐ Yes	☐ No
**More frequent airing**	☐ Yes	☐ No
**Face mask at home**	☐ Yes	☐ No

Other measures:

Have you been in quarantine again?

☐ Yes: ☐ once again ☐ several time ☐ No

Is your health currently impaired? Do you have any symptoms?

☐ Yes, the following:

☐ No

What is your current (personal) level of sickness?

(on a scale from 0-10, 0 = health not impaired, 10 = maximum impaired)

0 1 2 3 4 5 6 7 8 9 10

Not impaired Maximum impaired

Have you done a SARS-CoV-2 serology test?

☐ Yes, Reason for testing: ☐ No

Date:

Result: ☐ positive ☐ negative

Have you been vaccinated for SARS-CoV-2?

☐ Yes: How many injections? When? Which vaccine?

☐ No
